# An interview with Chris Chang

**DOI:** 10.1590/2177-6709.23.1.018-021.int

**Published:** 2018

**Authors:** Chris Chang

**Affiliations:** » PhD in bone physiology and Certificate in Orthodontics from Indiana University, in 1996. » Diplomate of American Board of Orthodontics(ABO). » Member of Angle Society of Orthodontists - Midwest. » Author of iAOI workbook, ABO Case Reports, Orthodontics, Jobsology and publisher of International Journal of Orthodontics and Implantology (iJOI). » Frequently lectures worldwide on a wide range of topics, including impaction treatment, OrthoBoneScrews, Orthodontic-Implant combined treatment and Jobs’ effective presentations. » As a private instructor since 2006, has taught over 2,000 doctors from more than 21 countries. » Founded Newton’s A, Inc. and Beethoven Orthodontic and Implant Group, based in Hsinchu, Taiwan. » Produced a complete series of video courses on orthodontics and implantology and the app Beethoven Dental Encyclopedia. » Has been actively involved in the design of orthodontic bone screws and its application on impaction treatment. » His latest focus is implant-orthodontic combined treatment.

Orthodontics has undergone a profound transformation since the skeletal anchorage became part of treatment plans. More specifically, orthodontic mini-implants presented by Kanomi in 1997 allowed us to achieve encouraging results when the anchorage subject is taken into account. Over the last decade, we have seen a revolution in the way we were accustomed to using the mini-screws allocated primarily in inter-radicular areas, that is, between the roots to reinforce anchorage during orthodontic mechanics. The revolution came just as a Taiwanese researcher showed the world how we could use the screws in an absolutely innovative and sophisticated way by installing them in areas previously unknown to most orthodontists: in extra-alveolar regions. Thus, the extra-alveolar mini-implants inserted in the regions of the infrazigomatic crest, mandibular ramus and buccal shelf appeared, allowing free dental movement along the maxillary and mandibular arches, since the screws are in areas outside the line of root action. With great honor I present to you in this interview one of the creators and “father” of this sophisticated system of anchorage using extra-alveolar screws, Dr Chris Chang. He has definitely modified the way we think today in the resolution of orthodontic problems in a rational and simple way, where the phrase *“It is easy, believe me!”* had never been so true as he presented. Orthodontics will never be the same after the introduction of this type of skeletal anchorage. I received with great satisfaction the mission to coordinate this interview with Prof Chang, which I had the privilege of knowing five years ago with his indispensable help in the elaboration of the first article^1^ published in Brazil on the subject of screws in buccal shelf to treat skeletal Class III malocclusion. Dr Chris Chang is one of the most expressive names in world Orthodontics, currently dividing his professional time in his private practice (Beethoven) where he attends to his patients and teaches several courses. Personal time he enjoys with his family, his dear wife and his two daughters, and their hobbies, which are golf and classic music. I hope that readers can take advantage of this interview granted to our beloved *Dental Press Journal of Orthodontics*. Marcio R. Almeida - interview coordinator 

A Ortodontia vem sofrendo uma profunda transformação desde que a ancoragem esquelética passou a fazer parte dos planos de tratamento. Mais especificamente, os mini-implantes ortodônticos, apresentados por Kanomi em 1997, permitiram alcançar resultados encorajadores quando o assunto ancoragem é levado em conta. Durante a última década, pudemos observar uma revolução na maneira que estávamos acostumados a usar os mini-implantes, inseridos prioritariamente em áreas interradiculares, para reforçar a ancoragem durante a mecânica ortodôntica. A revolução aconteceu exatamente a partir do momento em que um pesquisador de Taiwan mostrou ao mundo como poderíamos utilizar esses parafusos de maneira absolutamente inovadora e sofisticada, instalando-os em áreas até então desconhecidas pela maioria dos ortodontistas - em regiões extrarradiculares, ou extra-alveolares. Surgiram, assim, os mini-implantes extrarradiculares inseridos nas regiões de crista infrazigomática, ramo mandibular e buccal shelf, permitindo a livre movimentação dentária ao longo das arcadas superior e inferior, já que os mini-implantes encontram-se longe das raízes. Assim, é com imensa honra que apresento-lhes, nessa entrevista, um dos criadores e “pai” desse sofisticado sistema de ancoragem esquelética extra-alveolar, o Dr. Chris Chang, que modificou definitivamente a forma como pensamos na resolução biomecânica dos problemas ortodônticos, de uma maneira racional e simples, fazendo que a frase *“It is easy, believe me!”* soe mais verdadeira do que nunca. Uma coisa é certa, a Ortodontia nunca mais será a mesma depois da introdução desse tipo de ancoragem. Recebi com grande satisfação a missão de coordenar essa entrevista com o Prof. Chang, o qual tive o privilégio de conhecer há 5 anos, ao contar com sua imprescindível ajuda na elaboração do primeiro artigo^1^ publicado no Brasil sobre o tema de parafusos na buccal shelf para tratamento de Classe III esquelética. O Dr. Chris Chang, um dos mais expressivos nomes da Ortodontia mundial, atualmente divide o seu tempo profissional em sua clínica privada, onde atende seus pacientes e ministra diversos cursos. O tempo pessoal ele aproveita com a família, sua querida esposa e suas duas filhas, e com seus hobbies, que são o golfe e a música clássica. Espero que os amigos leitores possam aproveitar essa entrevista concedida à nossa querida revista Dental Press.

**Figure:**
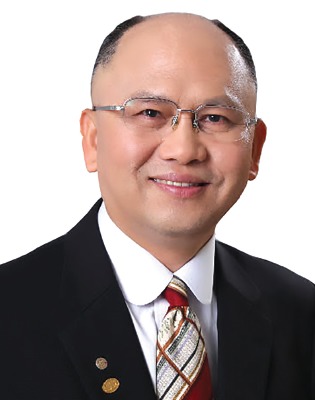

Dr. Chang, how the idea of using infrazygomatic (IZC) and buccal shelf (BS) screws started? Could you tell the history behind your famous protocol of using extra-alveolar screws? Marcio Almeida

Asian patients were reluctant to wear headgears and preferred non-surgical treatment for Class III, and this preference remains true till this day. So 15 years ago, together with Drs. John Lin and Johnny Liao, we started to study and modify Korean’s screw application method, in our study club, and began to place extra-alveolar screws. After one year of trial and error, we set up this protocol. In particular, I designed a screw with a rectangular hole around the neck for inserting a 3D lever arm, for dental impaction treatment. Using ramus screws to uncover lower horizontal impacted teeth is also an innovative approach to impaction treatment.

You seem very relaxed and secure in your lectures. Did this come naturally to you or did you have to work on it? How would you teach someone who wants to become a speaker as good as you are? Whose lectures did you enjoy watching most? Weber Ursi

If you have something to share with your friends and you speak from your heart, the rest is easy. Sharing is the key. Not showing off. Watching TED talks is a great way to start.

After traveling worldwide giving lectures, could you please make some comments on profession differences across the different countries that you have visited? Marcio Almeida

1. A higher percentage of patients in Asia require interdisciplinary treatment, which presents both challenges and opportunities for dentists in this region.
2. In terms of learning and teaching, I find the trend across the globe is leaning towards online and mobile learning whether it’s Orthodontics or other specialties.
3. I think the fastest growth in Orthodontics will be in Brazil, China, Russia, and the Middle East.

Your Class III cases treated with buccal shelf screws have demonstrated the possibility of the distal movement of the entire mandibular dentition. What is your success rate in this modality? Do you overcorrect? Which cases one could anticipate a higher risk of relapse? Weber Ursi

 My success rate is around 95%. 

 I routinely overcorrect 1-2 mm. 

 Those that have insufficient space between posterior molars and the border of ascending ramus have a higher risk of relapse, due to the lack of room for distalization.

What is the recurrence rate for the cases that apparently would require dental extractions and orthognathic surgery and that were treated without a surgical approach, with the aid of skeletal anchorage? In the presence of relapse in which retreatment requires surgical intervention, how the patient reacts? Do you, in the presence of bordeline cases, give prior warning that surgical intervention may be necessary? Matheus Pithon

The clinical judgement of whether to perform extractions and/or surgeries or not, in my view, is based on accurate diagnosis, reasonable treatment plans and most of all, the availability of appropriate treatment tools. I routinely inform patients of the pros and cons of each treatment plan and leave them to choose the preferred option. Having said that, my general treatment orientation is inclined to provide a minimally invasive care. In the case of relapse, I usually provide free retreatment as a punishment and a motivator for improvement.

Unbiased orthodontic literature has not shown that the Damon system is superior to conventional brackets. What are the reasons that made you start and continue using them even though scientific evidence apparently does not support most of the claims attributed to it? Weber Ursi

Although the currently available clinical research studies fail to provide a definitive answer to the superiority of the Damon system *versus* conventional brackets, my own extensive clinical experiences and natural instincts have convinced me it is. I’d also like to warn readers about the interpretation of these evidence-based research studies in Orthodontics. There are many variants that are hard or impossible to standardize or control in a clinical orthodontic research, such as the skill level of clinicians. As such, conclusions drawn from these types of research studies should be taken with a critical view. 

As a protocol for treatment of complex cases that would traditionally fall into dental extractions and/or orthognathic surgery, you use self-ligating brackets associated with extra-radicular implants. Do you attribute to these devices the success achieved or can we hypothetically achieve similar results with conventional brackets associated with extraoral headgear? Matheus Pithon

I find this combination of PSL (passive self-ligating) bracket system and extra-radicular TADs works pretty well in my current practice. I don’t think my old protocols could have achieved the same quality of results with equal efficacy and efficiency.

One of the problems of inserting a 12-mm length screw into IZC area is the possibility of hitting the sinus of the patient. Do you think that this really is a clinical problem? Marcio Almeida

With sufficient insertion torque, normally larger than 10Ncm, the success rate is very high. Sinus infection should not be a concern, in my experience.

What are your thoughts on the use of stainless steel versus titanium extra-alveolar screws? Are there any recommendations to use one instead of other? Marcio Almeida

I prefer manual insertion and this method requires stronger, more resilient screws. Therefore, I use screws of 2mm in diameter, made by stainless steel. While in maxilla the bone is relatively softer, both SS and titanium screws are equally applicable; SS screws are less likely to fracture in mandible, compared to titanium ones. Therefore, I only use SS screws in mandible.

In terms of success rate, there’s no statistical difference between SS and titanium screws. I’ve recently finished a study on this topic and expect to send it for publication in the near future.

Do you think that the understanding of “biomechanical concepts” is important while using IZC and BS screws? Could you provide us with some examples of the application of biomechanics in your clinical routine? Marcio Almeida

Most definitely. Please refer to my YouTube channel (search for “newtonsa0301”). I include biomechanics analysis in almost all cases I shared on this channel. 

It seems that our sequence of orthodontic wires used for leveling and aligning the teeth nowadays has changed over the last decade. Do you have any particular sequence of orthodontic wires as for the use of your extra-alveolar screws in your patients? Marcio Almeida

My wire sequence remains pretty much the same over the years, as shown bellow:

**Table:** 

0.014-in CuNiTi	4 months
0.014 x 0.025-in CuNiTi	4 months
0.017 x 0.025-in TMA	3 months
0.016 x 0.025-in SS	Until the end of treatment

You can start the screw mechanics in any stage. The guiding principle is light wire and light force.

For force level, use 8-12 oz for maxilla and 10-14 oz for mandible.

In a paper^2^ published in 2015, you showed us that an en-masse retraction of the mandibular arch is efficient for conservatively treating a skeletal Class III malocclusion. Posterior mandibular anchorage with buccal shelf screws causes intrusion of the molars to close the vertical dimension of the occlusion and rotation of the occlusal plane and the mandibular plane angle. Could you show us how this seems to work? Marcio Almeida

As the center of resistance of the whole mandible is located around the apex of the lower second premolar, the force will create a large counter clockwise rotation movement which results in rotating the occlusal plane. Please refer to my YouTube channel (search for “newtonsa0301”) for details.

You provide, on YouTube, your cases with great difficulty in orthodontic resolution, in which you present the treatment with the use of extra-radicular anchorage. The fact of being presented in an uncontrolled vehicle like YouTube could make orthodontists without solid training extrapolate the proposed protocol to cases with clear surgical indication? Matheus Pithon

It’s hard or close to impossible to control information on the internet in our time. My intention has always been sharing what I deem useful with anyone who is interested to learn. If you can think of any topic that may benefit the learning of all and hasn’t been shown on my channel (“newtonsa0301”), please feel free to let me know. 
